# CCR2 Deficiency Impairs Ly6C^lo^ and Ly6C^hi^ Monocyte Responses in *Orientia tsutsugamushi* Infection

**DOI:** 10.3389/fimmu.2021.670219

**Published:** 2021-07-05

**Authors:** Michael Petermann, Zacharias Orfanos, Julie Sellau, Mohammad Gharaibeh, Hannelore Lotter, Bernhard Fleischer, Christian Keller

**Affiliations:** ^1^ Department of Molecular Biology and Immunology, Bernhard Nocht Institute for Tropical Medicine, Hamburg, Germany; ^2^ Institute of Virology, University Hospital Giessen and Marburg, Marburg, Germany; ^3^ Department of Basic Veterinary Medical Science, Jordan University of Science and Technology, Faculty of Veterinary Medicine, Irbid, Jordan

**Keywords:** inflammatory monocytes, scrub typhus (Tsutsugamushi disease), chemokines, rickettsiosis, chemokine receptor (CCR2), interstitial pneumonia

## Abstract

*Orientia (O.) tsutsugamushi*, the causative agent of scrub typhus, is a neglected, obligate intracellular bacterium that has a prominent tropism for monocytes and macrophages. Complications often involve the lung, where interstitial pneumonia is a typical finding. The severity of scrub typhus in humans has been linked to altered plasma concentrations of chemokines which are known to act as chemoattractants for myeloid cells. The trafficking and function of monocyte responses is critically regulated by interaction of the CC chemokine ligand 2 (CCL2) and its CC chemokine receptor CCR2. In a self-healing mouse model of intradermal infection with the human-pathogenic Karp strain of *O. tsutsugamushi*, we investigated the role of CCR2 on bacterial dissemination, development of symptoms, lung histology and monocyte subsets in blood and lungs. CCR2-deficient mice showed a delayed onset of disease and resolution of symptoms, higher concentrations and impaired clearance of bacteria in the lung and the liver, accompanied by a slow infiltration of interstitial macrophages into the lungs. In the blood, we found an induction of circulating monocytes that depended on CCR2, while only a small increase in Ly6C^hi^ monocytes was observed in *CCR2^-/-^* mice. In the lung, significantly higher numbers of Ly6C^hi^ and Ly6C^lo^ monocytes were found in the C57BL/6 mice compared to *CCR2^-/-^* mice. Both wildtype and CCR2-deficient mice developed an inflammatory milieu as shown by cytokine and *inos*/*arg1* mRNA induction in the lung, but with delayed kinetics in CCR2-deficient mice. Histopathology revealed that infiltration of macrophages to the parenchyma, but not into the peribronchial tissue, depended on CCR2. In sum, our data suggest that in *Orientia* infection, CCR2 drives blood monocytosis and the influx and activation of Ly6C^hi^ and Ly6C^lo^ monocytes into the lung, thereby accelerating bacterial replication and development of interstitial pulmonary inflammation.

## Introduction

Scrub typhus is a mite-borne, neglected tropical infection caused by the obligate intracellular bacterium *Orientia (O.) tsutsugamushi*. In humans, transmission of *O. tsutsugamushi* during mite bites induces a cutaneous necrosis, the eschar (1), and is accompanied by undifferentiated fever in mild cases. In more severe cases, the infection may progress to interstitial pneumonia, acute respiratory distress syndrome (ARDS), myocarditis, encephalitis or other complications that may be lethal if not treated adequately.

*O. tsutsugamushi* has long been known to be a potent inducer of monocyte-related chemokines in humans and mice (2–4). Robust evidence supports a macrophage/monocyte tropism for this pathogen in blood and tissue (1, 5, 6) and strong macrophage responses in infected tissues (7, 8). Moreover, *O. tsutsugamushi* infects and replicates in human blood-derived monocytes *ex vivo*, as it does in neutrophils (9, 10).

In the pathogenesis of human scrub typhus, chemokine networks play an important role, and increased plasma concentrations of the chemokines CC chemokine ligand (CCL) 2 (monocyte chemoattractant protein-1), CCL4 (macrophage inflammatory protein-1β) and interleukin (IL)-8 have been associated with disease severity (11).

Chemokines shape the type and extent of monocyte responses. In mice, there are two major subsets of circulating monocytes: First, the “inflammatory” or classical monocytes, identified as CD11b^+^ Ly6C^hi^ Ly6G^-^ and expressing the CC chemokine receptor 2 (CCR2) at high levels. These inflammatory monocytes can be rapidly recruited into inflamed tissues. In mice lacking CCR2, a receptor for CCL2 and CCL7, severe defects in recruitment of inflammatory monocytes and tissue macrophages are observed (12–14). Consequently, CCR2 deficiency in mice drastically increases the susceptibility to infection with intracellular bacteria, viruses and protozoan parasites (13, 15–17). Inflammatory monocytes may, on the other hand, mediate pathology in inflammatory diseases such as atherosclerosis, where their absence confers an attenuated phenotype (12), or in the formation of liver abscesses in amebiasis (18).

A second subset of “resident” monocytes is identified as CD11b^+^ Ly6C^lo^ Ly6G^-^; these cells express only low levels of CCR2, but high levels of CX_3_CR1 (19). CD11b^+^ Ly6C^lo^ monocytes have a “patrolling” function in and around the vascular endothelium (20). They can rapidly extravasate into inflamed tissue where they follow a typical macrophage differentiation program. Subsequently, they may give rise to M2 type macrophages expressing *arg1*, e.g. in atherosclerosis (21). It was recently shown in a model of sterile hepatic inflammation that CCR2^hi^ monocytes are recruited to injured sites and transition *in situ* to CX3CR1^hi^ CCR2^lo^ monocytes (22).

Recent studies have revealed that there is a substantial degree of plasticity among macrophage subsets. Tissue-resident macrophages such as Kupffer cells, microglia, Langerhans cells and alveolar macrophages are now known to derive from embryonic precursors (23, 24), but circulating blood monocytes can replenish these macrophage populations upon tissue injury later in life (25, 26). Upon exposure to external stimuli, macrophage activation can follow either the “classical” M1 pattern, after exposure to bacteria or interferon (IFN)-γ (27, 28), or the “alternative” M2 pattern, after exposure to IL-4 (29, 30). With an increasing number of polarizing stimuli and subtypes of polarization identified, the M1/M2 paradigm has been challenged, and the need for immunological contextualization was highlighted (31, 32).

While the chemokine CCL2 is produced ubiquitously in many infections, it had long remained unknown whether this chemokine could act over a long distance, or if the bone marrow needed to be locally infected to induce the egress of monocytes. Shi et al. demonstrated that the first critical step is local CCL2 production in the bone marrow: mesenchymal stem cells produce CCL2 in response to very low levels of circulating Toll-like receptor (TLR) ligands and thus trigger release of Ly6C^hi^ CCR2^+^ monocytes into the bloodstream (33). Thereby, CCR2-mediated signals in bone marrow determine the frequency of Ly6C^hi^ monocytes in the circulation (14).

In the lung of *Orientia*-infected mice, interstitial and peribronchial macrophage lesions have been identified (7). Similarly, in the lung of human patients, the typical finding is an interstitial pneumonia, which seems to be associated with disease severity (34–36). While it was shown that *Orientia* drives M1 responses in the lungs (8), no mechanistic studies on chemokine-driven recruitment of myeloid cells have been provided yet. In the present study, we hypothesized that CCR2 is critical for the development of pulmonary monocyte/macrophage responses and associated bacterial clearance in *Orientia* infection. We reveal a role for CCR2 in formation of interstitial lung lesions, in shaping pulmonary and blood myeloid compartments and local pathogen defense, in a model of self-healing intradermal *Orientia* mouse infection.

## Methods

### Animal Experiments

All animal experiments were approved by the Hamburg Authority for Health and Consumer Protection (no. 106/15) and complied with the provisions of the Animal Welfare Act. CCR2-deficient mice on the C57BL/6 background, that were originally created on the 129/Ola background (37, 38), were kindly provided by Prof. Frank Tacke, Department of Hepatology and Gastroenterology, Campus Charité Mitte (CCM)/Campus Virchow-Klinikum (CVK), Charité, Berlin, and backcrossed >10 times to C57BL/6 mice.

Animals were bred and kept in the animal facility of the BNITM Hamburg in individually ventilated cages (IVCs). C57BL/6 wildtype mice were purchased from Charles River Laboratories (Sulzfeld, Germany). Animals were grouped of up to 4 animals per cage. Females were used at the age of 6-12 weeks. Infections with *O. tsutsugamushi* were performed in the BSL3 animal suit at BNITM, Hamburg. Pellet food and water were available to the animals *ad libitum*. Cages, bedding, cellulose paper for nest building, food and water were changed once a week.

### Infection of Animals

Infectious stocks of *O. tsutsugamushi* Karp-infected and uninfected L929 cells were stored in liquid nitrogen in 2 ml tubes (Nunc, Thermo Fisher Scientific). For infection, stocks were thawed, reconstituted in RPMI medium, washed twice, taken up in 1 ml PBS and stored on ice until intradermal injection in 1.5 ml Eppendorf tubes. Accordingly, L929 control cells were treated. Before infection, mice were anesthetized with 10 µl/g body weight of a solution containing ketamine (12 mg/ml) and xylazine (1.6 mg/ml). When the animals showed no reflexes, mice were infected intradermally over several sites of the right hind footpad (unilaterally), with a total dose of 5000 spot-forming units (*sfu*) (7).

### Collection of Organs

Prior to manipulation, mice received a lethal anesthesia, containing ketamine (12 mg/ml) and xylazine (1.6 mg/ml) at a weight-adapted dose of 15 μl/g body weight. When reflexes were absent, animals were dissected and organs removed. For preparation of lungs, the inferior vena cava was incised for rapid bleeding. The right cardiac ventricle was punctured with an 18G cannula. A button cannula was inserted into the ventricle *via* the puncture site and passed over the pulmonary valve. Subsequently, the lung was perfused through the pulmonary artery with 5 ml PBS. Finally, the lungs were freed of connective tissue and removed together with the proximal trachea. The liver was collected from the abdominal cavity. The organs were stored in 5 ml RPMI in 6-well plates on ice until further processing. Neck dislocation was performed immediately after organ removal. Blood samples collected from the vena cava were centrifuged at 6000 g for 5 minutes, and the serum was transferred to a 1.5 ml tube and stored at -20°C until further processing.

### Mandibular Blood Sampling for Flow Cytometry Analysis

By puncturing the submandibular plexus with a lancet, blood was taken using a heparinized capillary tube (20 μl) and immediately purged in 1 ml PBS-filled FACS tube using a 20 μl pipette. A tissue swab was applied onto the puncture site for 30 seconds to avoid possible rebleeding.

### Preparation of the Lung for Histology

To maintain the structure of the initially collapsed lungs, the airways were filled with 1% formalin. After irrigation of the pulmonary artery with 5 ml of PBS (Life Technologies, Darmstadt, Germany), the trachea was exposed by a cutaneous longitudinal incision from the thoracic aperture to the mandible and a straight cannula inserted distally between the cartilage clasps. The cannula was fixed with a thread, and the airways were filled *via* this access with 0.7 ml of 1% formalin solution, followed by closure of the trachea with the thread. The lung was removed from the thorax and transferred to a 15 ml tube filled with 10 ml of 1% formalin. All preparations were stored at 4°C until processing by the Mouse Pathology Facility at the Institute of Neuropathology (Prof. Dr. M. Glatzel, University Hospital Hamburg Eppendorf, Germany). Immunohistological staining for IBA1 (ionized calcium binding adapter molecule 1; WAKO, Neuss, Germany) was performed using the Ventana Benchmark XT (Ventana, Tuscon, Arizona, USA), as previously described (7). Sections were recorded with a BZ-9000 Keyence fluorescence microscope.

Co-staining of a lung sample from an *Orientia*-infected C57BL/6 mouse (retrieved on day 14 p.i.) for IBA1 (rabbit-anti-IBA1), *O. tsutsugamushi* (mouse-anti-56kD) and DAPI (Sigma, Darmstadt, Germany) was performed as previously described (7). Images were recorded with an Olympus confocal microscope.

### Quantification of Histological Images Using ImageJ

Images were imported into Adobe Photoshop, and areas devoid of tissue, representing empty air-filled space, were selected using the Magic Wand Tool (“Anti-alias” and “contiguous” deselected) using an appropriate tolerance value (usually between 12-20). Selected areas were deleted by menu: Edit > Clear. Resulting images, with empty space as white, were imported in ImageJ. Bronchial areas in images of the Parenchyma were manually erased, and vice-versa. Every image was analysed with the IHC Toolbox Plugin (https://imagej.nih.gov/ij/plugins/ihc-toolbox/index.html), scanning for brown color (representing IBA1 staining) with the H-DAB model. This returns a new separate image with all brown-stained areas extracted on a white background. Both images, the edited source and the extracted brown staining, were converted to monochromatic 8-bit, and an intensity threshold was applied (menu: Image > Adjust > Threshold), with the upper limit a few values below the pure white maximum. This represents every pixel that contains data as black and empty background pixels as white. The black area of both images, the one derived from the source image and the one from the IHC Toolbox scan, was measured by menu: Analyse > Measure. The ratio of the two measurements represents the percentage of the tissue area that is stained in the source image, with all air-filled areas excluded.

### Preparation of Blood Samples for FACS Analysis

Peripheral venous blood samples (20 µl) were resuspended in 1 ml of FACS buffer and then centrifuged for 5 min at 322xg and 4°C. The pellet was resuspended in 0.7 ml of erythrocyte lysis buffer (10% 0,17 M TRIS, 90% 0,17 M NH_4_Cl pH 7,4), and incubated for 6 minutes at room temperature. The now translucent suspension was diluted with 2 ml of FACS buffer to stop the lysis, and centrifugation was repeated.

### Single Cell Suspensions From Lung

The lungs were cut into small pieces using a scalpel, in sterile Petri dishes on ice. The tissue pieces were transferred into 15 ml tubes filled with 3 ml of DNAse (Sigma-Aldrich, Deisenhofen, Germany)/collagenase D (Roche diagnostics, Risch, Switzerland) solution (1:100 collagenase stock solution; 10 μg/mL DNAse I) and incubated in a 37°C water bath for 60 minutes. Every 20 minutes, the samples were sheared 3 times with a sterile Pasteur pipette. After one hour, the enzyme activity was stopped by addition of 150 μl 0.1 M EDTA (Sigma-Aldrich, Deisenhofen, Germany) sheared again, and incubated for 5 more minutes at 37°C. The tissue samples were then passed *via* a 70 μm Cell Strainer (Becton Dickinson, Heidelberg, Germany) using 2 ml RPMI into a 50 ml tube.

The suspension was centrifuged at 322xg/4°C for 5 minutes and the supernatant discarded. After two more washes with 5 ml of RPMI, the suspensions were taken up in 0.5 ml of RPMI, and leucocytes were counted in a Neubauer counting chamber (Hecht-Assistant, Sondheim, Germany).

### Flow Cytometric Analysis

Antibody staining of cell suspension from blood or lungs was done in 5 ml tubes (Sarstedt, Nümbrecht, Germany). Centrifugation was carried out at 322 x g and 4°C for 5 minutes unless otherwise specified. For lungs, 10^8^ cells were transferred to one tube. From blood samples, all cells were used. Cells were washed in 2 ml FACS buffer, and incubated with 50 μl Fc-block (BNITM, Hamburg, Germany) for 10 minutes at 4°C. Subsequently, 50 μl of prediluted antibodies (diluted in Fc-block) were added and incubated for 60 minutes at 4°C in the dark. After incubation, cells were washed with 2 ml of FACS buffer. Cells were fixed with 100 μl of 4% paraformaldehyde (Serva, Heidelberg, Germany) in PBS and incubated for 20 minutes at 4°C in the dark. Intermediate storage took place at 4°C in the dark.

Antibodies used for blood panel: FITC-anti-Ly-6C (clone: HK1.4, BioLegend, San Diego, CA, USA); PE-anti-MHCII (clone: M5/114.15.2, Ebioscience); PerCP-Cy5.5-anti-CD11b (clone: M1/70, BD); APC-anti-Ly6G (clone: 1A8, BioLegend). Antibodies used for lung/spleen panel: eFluor^®^450-anti-CD11b (clone: M1/70, Ebioscience); FITC-anti-MHCII (clone: M5/114.15.2, Ebioscience); PE-anti-Ly6C (clone: HK1.4, BioLegend) or BV510-anti-Ly6C (clone: HK1.4, BioLegend); PerCP-Cy5.5-anti-Ly6G (clone: 1A8, BioLegend); APC-anti-F4/80 (clone: BM8, Ebioscience). Antibodies used to measure CCR2 expression: BV510-anti-CD11b (clone: M1/70, Biolegend); FITC- or PE-anti-Ly6C (clone: HK1.4, BioLegend); APC/Cy7-anti-Ly6G (clone: 1A8, BioLegend); AF700-anti-CCR2 (clone: #475301, R&D).

All samples were analyzed by FACS within 12 hours of fixation. The antibody-labeled cell samples were run on a LSRII. Data analysis was carried out using FlowJo™ 10.06 or 10.07 software (Becton Dickinson; Ashland, Oregon, USA).

In order to quantify the concentration of leukocytes in single cell suspensions from organs, trypan blue-negative cells were counted manually in Neubauer chambers with an inverse light microscope. The entire processed sample - corresponding to 20 µl blood or one lung wing, and resuspended in 200 µl FACS buffer - was analyzed by LSRII; from the “cell gate”, the single cell count was then determined using FlowJo software. In case of blood, since 20 µl of blood samples were processed, the cell count obtained was divided by 20 to express results as single cells/µl blood. Using FlowJo, the frequency of each cell population (in %) with respect to all single cells was analyzed and multiplied with the single cell count.

For identification of CD11b^+^, Ly6G^+^, Ly6C^hi^ or Ly6C^lo^ populations, the gates were drawn in analogy to previously published reports (14, 39) and considering negative populations in the same sample.

### Isolation of DNA and Quantitative Real-Time PCR

Bacterial concentrations in samples from lung (50 mg), liver and spleen (30 mg) were determined by quantitative *traD* qPCR analysis, normalized to the DNA content of the eluate, as described (7). *TraD* qPCR reactions were performed on a 384-well LightCycler 480 (Roche, Mannheim, Germany).

### Statistical Analysis

Graphpad Prism 7.0 software was used for statistical analysis. Descriptive statistics show mean +/- SD. Hypotheses were tested by two-tailed t test, by one-way or two-way analysis of variance (ANOVA) with Bonferroni post-correction, or by Mantel-Cox test. A p value of <0.05 was considered significant.

### Isolation of mRNA

50 mg of lung tissue were transferred to 100 ml of Trizol (Invitrogen, Karlsruhe, Germany) in 2 ml Precellys tubes (Precellys, Bertin Technologies, Villeurbanne, France) and kept on ice. The tissue was homogenized (Precellys 24, Peqlab, Erlangen, Germany) and immediately frozen at -80°C. For phase separation, the frozen samples were thawed and centrifuged at 11800 x g for 5 min at 4°C. The supernatant was mixed with 0.2 ml of chloroform (Roth, Karlsruhe, Germany) and shaken manually for 15 seconds, followed by centrifugation at 11800 x g/15 min/4°C. The aqueous phase was mixed with 500 µl of isopropanol (Roth, Karlsruhe Germany). After incubation at room temperature (10 min) and centrifugation, (11800 x g/10min/4°C), the pellet was washed and vortexed with 75% ethanol (Roth, Karlsruhe, Germany). After centrifugation (7280 x g/5 min/4°C), the pellet was dissolved in 150 μl RNase-free water (Qiagen, Hilden, Germany) and incubated at 60°C for 12 minutes. The subsequent storage took place at -80°C. DNA digestion with DNAse (RNase-free DNase-Set, Qiagen, Hilden, Germany) was performed before RNA extraction (Rneasy Mini Kit, Qiagen, Hilden, Germany).

For this purpose, 85 µl DNA extract was mixed with 10 µl RDD buffer (DNAse-Set, Qiagen), then 2.5 µl DNAse-stock solution was added and incubated for 10 min at room temperature. Following the Qiagen RNeasy mini kit protocol, the sample was mixed with 350 µl RLT buffer or before 250 µl ethanol 70% was added and the mixture was placed on a column. Centrifugation was carried out at 8000 x g for 15 s at room temperature. The flow was discarded. Then 500 μl of RPE buffer was added over the column and centrifuged for 15 seconds at 8000 × g and room temperature. Another 500 µl of RPE buffer was added and centrifuged for 2 min at 8000 x g and room temperature. The column was placed in a new collection tube and again for a 1 min at the above. conditions centrifuged. the column was then placed in a 1.5 ml safe lock and 50 µl of nuclease-free water was added, again centrifugation for 1 min under the same conditions.

### Reverse Transcription and Quantitative Real-Time PCR

The nucleic acid concentration was determined by spectrophotometry (Nanodrop, ThermoFisher Scientific, Waltham MA, USA), and the sample was diluted for reverse transcription performed by using 2 µg RNA diluted in 10 µl nuclease-free water for further analysis. The high-capacity cDNA reverse transcription kit (Life Technologies, Waltham MA, USA) was used for reverse transcription in a Thermocycler (Primus advanced, Peqlab, Erlangen, Germany) of 10 µl RNA sample as recommended by the manufacturer (25°C, 10 min; 37°C, 120 min; 85°C, 5 min, storage at 4°C).

Gene expression analysis by quantitative real-time PCR was carried out on a 384-well LightCycler 480II (Roche Diagnostics, Risch, Switzerland). The 10 µl reactions (HotStar Taq, Qiagen, Germany) contained 1 µl cDNA template in the presence of 300 nM sense and antisense primers (see **Table 1**), 1mM additional MgCl_2_, 200 mM dNTPs, 0.25 U taq polymerase and 0.1 µl SYBR Green 1:1000 in DMSO (Sigma, Germany). Reaction condition were 95°C, 15 min; touchdown (6 cycles): 95°C, 30 s - 64>58°C, 40 s - 72°C, 30 s; amplification (29 cycles): 95°C, 30 s - 58°C, 40 s, 72°C, 30 s; melting curve: 95°C, 1 min - 67°C > 95°C [1°C/step] - 40°C, 20 s. Samples were run as technical duplicates; when not meeting a Cp standard deviation of <1, the measurement was repeated.

**Table 1:** Primers for gene expression analysis.

Target gene	Primer sequence	Source
***arg1* s (sense)**	5’-TCCAGAAGAATGGAAGAGTCAG-3’	this study
***arg1* as (antisense)**	5’-CAGATATGCAGGGAGTCACC-3’	this study
***il-4* s**	5’-GCATTTTGAAGAGGTCACAGG-3’	(40)
***Il-4* as**	5’-TATGCGAAGCACCTTGGAAGC-3’	(40)
***inos* s**	5’-TGGTGGTGACAAGCACATTTG-3’	(18)
***inos* as**	5’-AAGGCCAAACACAGCATACC-3’	(18)
***ccl2* s**	5’-TCTCTCTTCCTCCACCACCA-3’	(18)
***ccl2* as**	5’-CGTTAACTGCATCTGGCTGA-3’	(18)
***ifn-γ* s**	5’- GATGCATTCATGAGTATTGCCAAGT -3’	(18)
***ifn-γ* as**	5’- GTGGACCACTCGGATGAGCTC -3’	(18)
***tnf-α* s**	5’-GTTTGCTACGACGTGGGCT-3’	(18), modified
***tnf-α* as**	5’-CCAAATGGCCTCCCTCTCA-3’	(18), modified
***rps9* s**	5’- CCGCCTTGTCTCTCTTTGTC -3’	(18)
***rps9* as**	5’- CCGGAGTCCATACTCTCCAA -3’	(18)

### Gene Expression Analysis

The induction of gene expression was calculated as n-fold induction using the Cp-value of the respective target genes and the reference gene *rps9*. The following formula was used:

$$\frac{2_{}^{\left(\text{Cp target }[\text{non}-\text{infected}\;\text{control}]\;-\text{ Cp target}\;[\text{infected},\;\text{day}\;\text{x}]\right)}}{2_{}^{\left(\text{Cp }rps9\;[\text{non}-\text{infected}\;\text{control}]\;-\text{ Cp }rps9\;[\text{infected},\;\text{day}\;\text{x}]\right)}}$$

The efficiency of target and reference amplification was set to 2, and as controls, the average of mean Cp (both target and *rps9*) from non-infected animals (day 0) was used.

### Scoring of Mice

Mice were weighed using a digital scale (Denver-Instrument, Göttingen, Germany). The scoring was based on clinical and behavioral symptoms: 1: neck fur ruffled, loss of curiosity; 2: back fur ruffled, acute tiredness; 3: complete fur ruffled, heavy breathing, distended abdomen, apathy, hunched posture.

## Results

### IBA1^+^ Macrophages Are Host Cells for *O. tsutsugamushi* in the Lung

**Figure 1:**
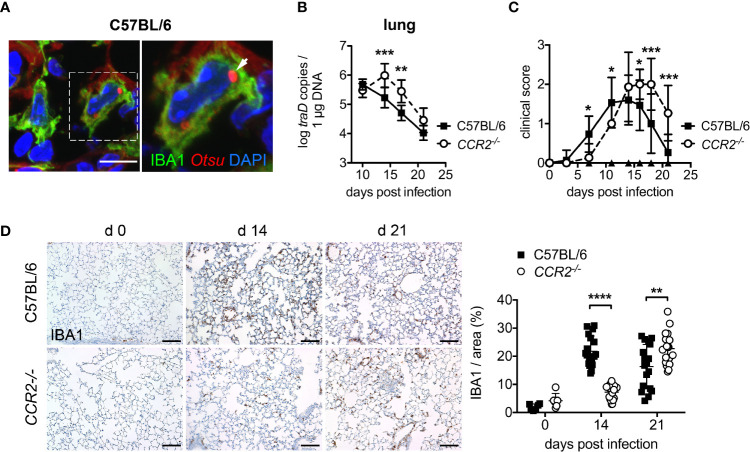
CCR2 deficiency delays bacterial clearance of *O. tsutsugamushi* and macrophage infiltration in the lung. **(A)** Immunofluorescence staining for *O. tsutsugamushi* (*Otsu*, anti-56 kD mAb, red) and macrophages (IBA1, green) from the lungs of an infected C57BL/6 mouse (day 14 p.i.) reveals intracellular *Orientia* organisms. Blue, DAPI. Scale bar: 10 µm. **(B)** *CCR2^-/-^* or C57BL/6 mice were infected with 5,000 sfu *O. tsutsugamushi* in the hind footpad. Per genotype, n=3 mice were sacrificed on days 10, 14, 17 and 21, and bacterial concentrations were quantified from the lungs by *traD*-specific qPCR. Data from two independent experiments were pooled; shown are means of bacterial concentrations from organs of n=2x3 = 6 mice per genotype and time point ± SD. **(C)** *CCR2^-/-^* or C57BL/6 mice (n=5 mice per genotype) were infected with 5,000 sfu *O. tsutsugamushi* in the hind footpad. Clinical scores were recorded on days 0, 3, 7, 11, 14, 16, 18 and 21. Shown are pooled results from three independent experiments (means of n=15 infected animals per group ± SD). **(D)** *CCR2^-/-^* or C57BL/6 mice were infected with 5,000 sfu *O. tsutsugamushi* in the hind footpad. N=3 mice per genotype and timepoint were sacrificed on days 14 and 21, and macrophages were stained by immunohistochemistry for IBA1 in histological sections from lungs. Representative histological images are shown from parenchyma areas. Scale bar: 100 µm. The IBA1^+^ area was quantified (6 areas per lung section from n=3 infected [day 14, 21] and n=1 uninfected [day 0] mice per genotype; mean ± SD). *p < 0.05; **p < 0.01; ***p < 0.001; ****p < 0.0001 by two-way ANOVA.

Macrophages, which can be histologically identified by IBA1 (ionized calcium binding adapter molecule 1)-specific staining (41), represent a major component of induced pulmonary infiltrates in mouse infections with *Orientia* (7, 42). In the lungs of infected mice, we identified intracellular *Orientia* in IBA1^+^ macrophages (**Figure 1A**). Yet, the mechanisms of macrophage recruitment to the lung, as well as their roles in bacterial dissemination and antibacterial defense remains unknown in *Orientia* infection.

### *CCR2^-/-^* Mice Show Delayed Bacterial Clearance, Symptom Development and Macrophage Invasion Into Lungs

The C-C chemokine receptor CCR2 is known to be vital for the recruitment of macrophages to inflamed tissues (13), *via* mobilization of inflammatory monocyte precursors from the bone marrow to the blood (14). Thus, we investigated whether CCR2-dependent recruitment of macrophages and monocytes is required for systemic dissemination of *O. tsutsugamushi* and protection. First, we assessed bacterial dissemination to lung and liver, clinical course and lung histopathology in CCR2-deficient and C57BL/6 wild type mice. The bacterial concentrations on day 10 p.i. in lungs (**Figure 1B**) and liver (**Figure S1**) did not differ between *CCR2^-/-^* and wildtype mice, suggesting an unimpeded dissemination from the cutaneous inoculation site to internal organs. However, *CCR2^-/-^* mice had higher bacterial concentrations on day 14 and 17 p.i. in both organs, suggesting an impaired clearance of *O. tsutsugamushi* in the absence of CCR2-dependent inflammatory monocytes (**Figures 1B**, **S1**). Also, *CCR2^-/-^* mice showed delayed onset and delayed recovery from symptom development (**Figure 1C**). This delay in the course of symptoms and bacterial clearance in *CCR2^-/-^* mice also correlated with a delayed pulmonary influx of IBA1^+^ macrophages to the lungs (**Figure 1D**). Thus, bacterial clearance, onset and recovery from symptoms and pulmonary macrophage inflammation (as investigated by histopathology) were accelerated by a CCR2-dependent cell population in our model of *O. tsutsugamushi* infection.

### Defective Mobilization of Ly6C^hi^ and Ly6C^lo^ Blood Monocytes in *CCR2^-/-^* Mice During *O. tsutsugamushi* Infection

**Figure 2:**
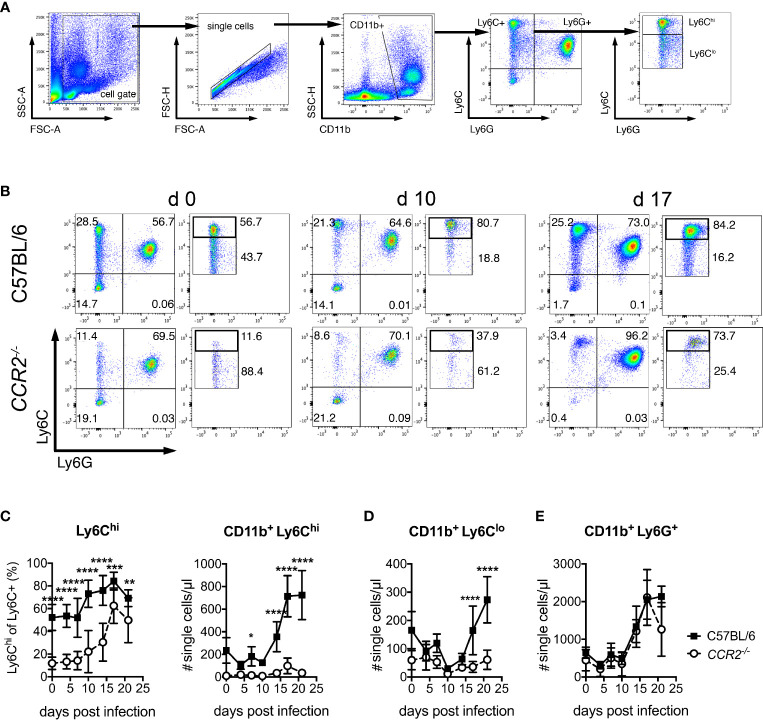
Induction of Ly6C^hi^ and Ly6C^lo^ monocytes in peripheral blood depends on CCR2 during *O. tsutsugamushi* infection. C57BL/6 and *CCR2^-/-^* mice were infected i.d. with *O. tsutsugamushi*. From venous blood samples retrieved from infected animals on days 4, 7, 10, 14, 17 and 21 p.i., peripheral blood mononuclear cells were stained for CD11b, Ly6C and Ly6G and analyzed by FACS. **(A)** Gating strategy for blood neutrophils and monocyte subsets. **(B)** Analysis of Ly6C and Ly6G expression on CD11b^+^ cells. For Ly6C^hi^ and Ly6C^lo^ cells, percentages are expressed as % of pre-gated CD11b^+^ Ly6C^+^ cells. Representative dotplots from d 0, d 10 and d 17 are shown. **(C)** Percentages of Ly6C^hi^/Ly6C^+^ cells (left) and concentrations of CD11b^+^ Ly6C^hi^ cells per µl blood. **(D)** Concentrations of CD11b^+^ Ly6C^lo^ and **(E)** CD11b^+^ Ly6G^+^ cells per µl blood at the indicated days p.i. Shown are pooled results from three independent experiments (mean ± SD), with n=4 animals per experiments (values from n=12 animals per genotype and time point). *p < 0.05; **p < 0.01; ***p < 0.001, ****p < 0.0001 by two way-ANOVA.

We next investigated whether *O. tsutsugamushi*-infected *CCR2^-/-^* mice show defective mobilization of inflammatory monocytes to the blood. To that end, the expression of CD11b, Ly6C and Ly6G on PBMCs was measured by FACS (for gating strategy see **Figure 2A**). To identify inflammatory monocytes, we analyzed the percentage of Ly6C^hi^ cells among all CD11b^+^ Ly6C^+^ cells and enumerated them (**Figures 2B, C**). Prior to infection, wildtype animals had an about 5-fold higher percentage of Ly6C^hi^ cells, compared to CCR2-deficient mice (**Figures 2B, C**). This percentage increased in wildtype mice from day 10 on, to about 80% on day 17 p.i. (**Figure 2C**). Interestingly, *CCR2^-/-^* mice also displayed an albeit reduced increase in their percentage of Ly6C^hi^/Ly6C^+^ monocytes in blood (**Figures 2B, C** left panel). Higher numbers of Ly6C^hi^ inflammatory monocytes were measured from day 14 p.i. in wildtype mice, giving rise to a 3-4-fold induction in blood in absolute numbers (**Figure 2C**, right panel). Notably, despite a drop in percentage (**Figures 2B, C** left panel), we also observed an induction of absolute numbers of CD11b^+^ Ly6C^lo^ monocytes from day 17 p.i. that was CCR2-dependent (**Figure 2D**). Contrarily, the induction of CD11b^+^ Ly6G^+^ neutrophils from day 14 p.i. was independent of CCR2 (**Figure 2E**).

Thus, wildtype mice showed an induction of both CD11b^+^ Ly6C^hi^ and CD11b^+^ Ly6C^lo^ monocytes in blood during infection with *O. tsutsugamushi* in C57BL/6 mice. In *CCR2^-/-^* mice, contrarily, a very small and delayed induction that was demonstrable only in the relative amount of Ly6C^hi^/Ly6C^+^ monocytes was found. Blood monocytosis during *O. tsutsugamushi* infection is thus largely CCR2-dependent.

### Reduced Influx of Ly6C^hi^ Monocyte Subsets Into the Lung of *CCR2^-/-^* Mice During *O. tsutsugamushi* Infection

**Figure 3:**
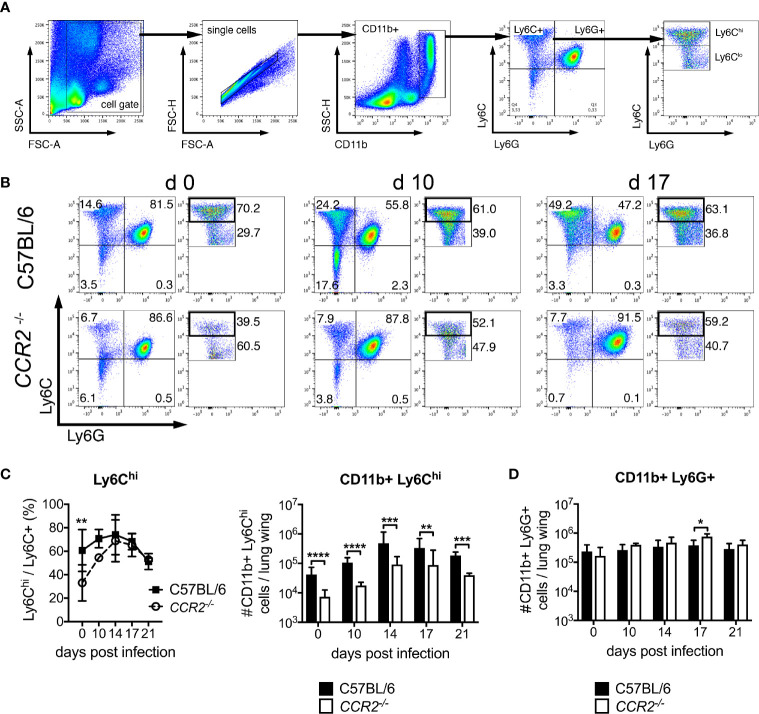
CCR2 drives expansion of Ly6C^hi^ monocyte but not granulocyte populations in the lung during infection with *O. tsutsugamushi*. CCR2-deficient or C57BL/6 mice were infected with 5,000 sfu *O. tsutsugamushi* in the hind footpad. On the indicated days post infection, animals were sacrificed (n=3 per genotype and timepoint), and pulmonary cells were analyzed for expression of CD11b, Ly6C and Ly6G by FACS. **(A)** Gating strategy for pulmonary neutrophils (Ly6G^+^) and monocyte (Ly6C^+^) subsets. **(B)** Representative dotplots from d 0, d 10 and d 17 p.i. **(C)** Percentages of Ly6C^hi^/Ly6C^+^ cells (left) and absolute numbers of CD11b^+^ Ly6C^hi^ cells per lung wing (right). **(D)** Absolute numbers of CD11b^+^ Ly6G^hi^ cells per lung wing. Shown are pooled results from two independent experiments, i.e. from a total of n=6 mice per genotype and timepoint; mean ± SD. *p < 0.05; **p < 0.01; ***p < 0.001, ****p < 0.0001 by two way-ANOVA.

Histopathology of lung samples suggested at delayed influx of macrophages to the lung in CCR2 deficiency (**Figure 1D**). In order to phenotypically and quantitatively characterize pulmonary inflammation, we analyzed Ly6C^hi^, Ly6C^lo^ and Ly6G^+^ cells (gating strategy in **Figure 3A**). In C57BL/6 mice, the percentage of Ly6C^hi^ among all CD11b^+^ Ly6C^+^ cells was significantly higher compared to *CCR2^-/-^* mice on day 0, but not during *Orientia* infection: from day 10 p.i., *CCR2^-/-^* mice displayed similar percentages of Ly6C^hi^ cells in the lung (**Figures 2B, C** right panel). With view to absolute numbers, however, we found clear differences between the two genotypes: C57BL/6 mice showed a much stronger induction to 4-8-fold higher numbers of Ly6C^hi^ monocytes in their lungs compared to *CCR2^-/-^* mice, peaking at day 14 p.i. (**Figure 3C**, right panel). Over the entire course of *Orientia* infection, these differences were highly significant. Also, >90-95% of the CD11b^+^ Ly6C^hi^ cells of both genotypes expressed F4/80, suggesting that the vast majority of this population were macrophages (**Figure S2A**). The expression of MHCII by CD11b^+^ Ly6C^hi^ cells was between 10-20% in uninfected mice and rose to >80-90% during infection, and we observed a faster upregulation of MHCII in C57BL/6 mice compared to *CCR2^-/-^* mice on day 10 p.i. (**Figure S2B**).

Contrarily, the numbers of neutrophils in the lung did not differ between both genotypes on days 0, 10, 14 and 21 p.i.; they increased comparably and were only slightly higher in *CCR2^-/-^* mice on day 17 p.i. (**Figure 3D**). These data suggest that recruitment of CD11b^+^ Ly6C^hi^ monocytes/macrophages to the lung requires CCR2. CCR2 however did not contribute to the recruitment of neutrophils to the lung.

### Impaired Pulmonary Ly6C^lo^ Monocytes in the Lung of *CCR2^-/-^* Mice During *O. tsutsugamushi* Infection

**Figure 4:**
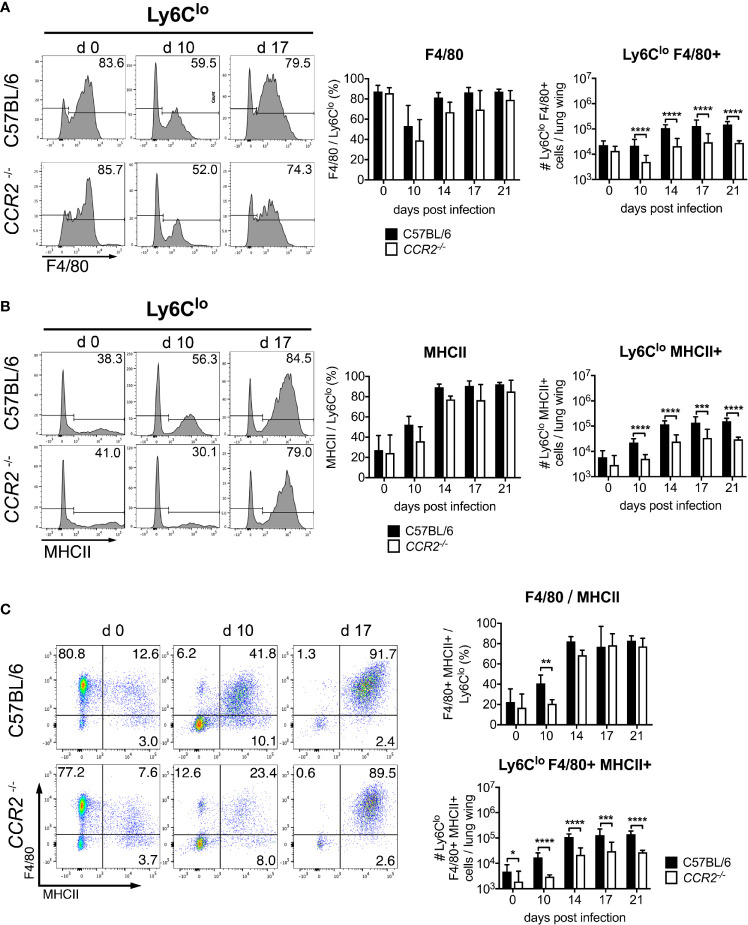
CCR2 is required for efficient recruitment of activated Ly6C^lo^ monocytes to the lung during infection with *O. tsutsugamushi*. *CCR2^-/-^* or C57BL/6 mice were infected as in **Figure 3**. On CD11b^+^ Ly6C^lo^ cells from the lung, the expression of F4/80 **(A)** and MHCII **(B)** was measured by FACS. Shown are representative histograms from d 0, d 10 and d 17 p.i. (left panels), percentages of marker-positive Ly6C^lo^ cells (middle panels) and absolute numbers per lung wing (right panels). **(C)** Co-expression of F4/80 and MHCII on CD11b+ Ly6C^lo^ cells. Representative dotplots from d 0, d 10 and d 17 (left), percentages (upper right) and absolute numbers per lung wing (lower right panel). Data were pooled from 2 independent experiments (n=6 mice per genotype and time point, mean ± SD). *p < 0.05; **p < 0.01; ***p < 0.001, ****p < 0.0001 by two way-ANOVA.

We also observed a prominent Ly6C^lo^ population in the CD11b^+^ Ly6C^+^ gate. Hypothesizing that these cells were also mainly macrophages, we investigated expression of the macrophage marker F4/80. Between 80-90% of pre-gated Ly6C^lo^ cells expressed F4/80 prior to infection (**Figure 4A**), suggesting a macrophage population. The percentage decreased to 40-50% on day 10 p.i., but increased again to 70-80% after day 14 p.i. Percentages of F4/80^+^ cells did not differ significantly between C57BL/6 and *CCR2^-/-^* mice (**Figure 4A**, middle panel). While the absolute numbers of pulmonary CD11b^+^ Ly6C^lo^ F4/80^+^ macrophages were comparable in both genotypes on day 0, they increased significantly in C57BL/6 mice on days 10, 14, 17 and 21 p.i. compared to *CCR2^-/-^* mice (**Figure 4A**, right panel). MHCII expression was found on 20-40% of Ly6C^lo^ cells on day 0, but the percentage increased to about 80-90% on days 14, 17 and 21 p.i. (**Figure 4B**, left and middle panels), suggesting an activated macrophage phenotype. Again, significantly higher numbers of CD11b^+^ Ly6C^lo^ MHCII^+^ cells were found in C57BL/6 mice (**Figure 4B**, right panel).

A co-expression analysis showed that during *Orientia* infection, MHCII was mostly expressed by F4/80^+^ cells (**Figure 4C**, left panels). Also, the F4/80/MHCII double-positive macrophage population increased earlier in wildtype mice. Again, *CCR2^-/-^* mice did not have a defect in upregulating expression of F4/80 and MHCII on Ly6C^lo^ cells (**Figure 4C**, upper right panel), but the population was 5-10-fold larger in wildtype mice compared to *CCR2^-/-^* mice (**Figure 4C**, lower right panel).

Although it is possible that the Ly6C^lo^ monocytes developed from the highly CCR2-expressing Ly6C^hi^ monocytes, as previously suggested (22, 43), it is also possible that their recruitment occurred directly *via* CCR2-dependent mechanisms. To confirm observations that Ly6C^lo^ cells also do express CCR2 (44), we measured expression of CCR2 on Ly6C^hi^ and Ly6C^lo^ blood monocytes (**Figures S3A–D**). Ly6C^lo^ monocytes of C57BL/6 mice had a higher MFI in CCR2 staining than cells from *CCR2^-/-^* mice or unstained controls, demonstrating their expression of CCR2 at low levels (**Figures S3C, D**).

Thus, efficient recruitment of both Ly6C^hi^ and Ly6C^lo^ monocyte/macrophages to the lung of *Orientia*-infected mice required CCR2. Contrarily, there was no defect in the influx of Ly6G^+^ neutrophils in *CCR2^-/-^* mice. The defect in CCR2-dependent monocyte/macrophage recruitment paralleled the impaired bacterial clearance in *CCR2^-/-^* mice.

### CCR2 Is Neither Required for Efficient Expansion of Ly6C^hi^ and Ly6C^lo^ Populations nor Bacterial Clearance in the Spleen

The induction of a small population of Ly6C^hi^ monocytes in the blood and the influx of some Ly6C^hi^ and Ly6C^lo^ monocytes/macrophages to the lungs of *CCR2^-/-^* mice was surprising, suggesting a CCR2-independent source of these cells. It was shown that the spleen can serve as an alternative reservoir for Ly6C^hi^ monocytes, derived from local progenitors, that can be deployed to distant sites such as the heart (45, 46). We therefore investigated the Ly6C^hi^ monocyte population in the spleen of *Orientia*-infected mice. A difference in percentages of Ly6C^hi^/Ly6C cells and in the size of the Ly6C^hi^ monocyte population was found only prior to infection: During *Orientia* infection, as assessed on days 10, 14, 17 and 21 p.i., *CCR2^-/-^* mice showed no significant differences to the wildtype regarding the percentages of Ly6C^hi^/Ly6C^+^ cells, and they also expanded absolute numbers of CD11b^+^ Ly6C^hi^ cells to the same extent as C57BL/6 mice (**Figures S4A, B**). Interestingly, the percentage of Ly6C^hi^ cells dropped more rapidly than in the lung, from about 80% on day 14 p.i. to about 40% on day 21 p.i. This was associated with a normal clearance of *Orientia* from the spleen of *CCR2^-/-^* mice as shown by qPCR, with no differences to the C57BL/6 wildtype (**Figure S4C**). Also, no gross defects in the size of Ly6C^lo^ monocyte and Ly6G^+^ neutrophil populations were observed in *CCR2^-/-^* mice (**Figures S4D, E**).

Thus, a normal expansion of Ly6C^hi^ monocytes in the spleen of *CCR2^-/-^* mice showed that recruitment of Ly6C^hi^ monocytes *via* CCR2 from the bone marrow to the spleen was not required, in contrast to what we observed in the lung (**Figures 3** and **4**). This expanded Ly6C^hi^ population could potentially constitute an alternative source for monocytes released to the blood or recruited to the lung.

### The Establishment of an Inflammatory Cytokine Milieu in the Lung Is Delayed in *CCR2^-/-^* Mice

**Figure 5:**
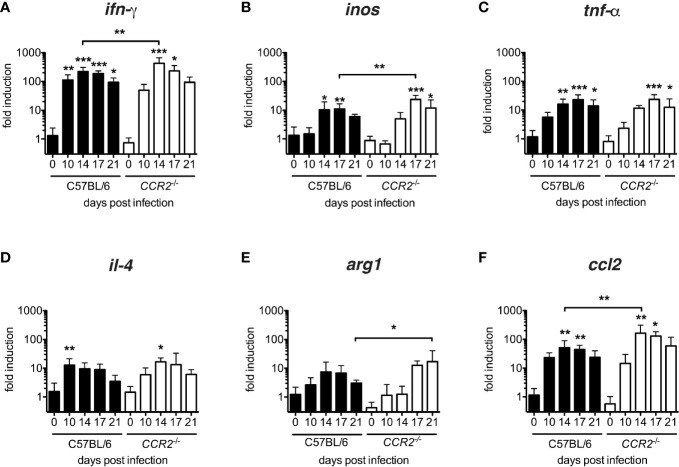
Delayed development of inflammatory milieu in lungs of *O. tsutsugamushi CCR2^-/-^* mice. *CCR2^-/-^* and C57BL/6 mice were infected with 5,000 sfu *O. tsutsugamushi* i.d. and sacrificed on days 0, 10, 14, 17 and 21 p.i. From homogenates of lung tissue, transcription of *ifn-γ*, *inos* and *tnf-α*, *il-4*, *arg1* and *ccl2* mRNA was measured by real-time PCR from tissue lysates and expressed as -fold induction at the indicated time points p.i. **(A–F)**. Shown are pooled results from two independent experiments (total of n=6 mice per genotype and timepoint; mean ± SD). *p < 0.05; **p < 0.01; ***p < 0.001 by one-way ANOVA.

In order to characterize the cytokine milieu in lung tissue, we measured transcription of *inos*, *tnf-α*, *ifn-γ* (considered as M1-associated genes) and *il-4* and *arg1* (considered as M2-associated genes) as well as *ccl2* mRNA by quantitative real-time PCR. All genes were up-regulated in both genotypes (**Figure 5**). However, a significant induction of IFN-γ with respect to the baseline transcription level was delayed from day 10 p.i. to day 14 (**Figure 5A**), in *inos* and *tnf-α* from day 14 to day 17 (**Figures 5B, C**) in *CCR2^-/-^* mice. Interestingly, M2-associated genes were also induced in both genotypes: Again, the peak of *il-4* transcription was delayed from day 10 to day 14 p.i. (**Figure 5D**) in *CCR2^-/-^* mice, and the peak of *arg1* transcription was even delayed from day 14 p.i. to day 21 p.i. (**Figure 5E**). In general, transcription of *ifn-γ*, *inos* and *ccl2* mRNAs were significantly increased in *CCR2^-/-^* mice on single days indicated (**Figures 5A, B, F**). This suggests that the development of an inflammatory cytokine milieu is delayed by several days in the absence of CCR2. The delay was consistent with the delayed influx of macrophages to the lungs (**Figures 1**, **3**).

### CCR2 Drives Early Infiltration of Macrophages to Lung Parenchyma, but Not Accumulation of Macrophages in BALT

**Figure 6:**
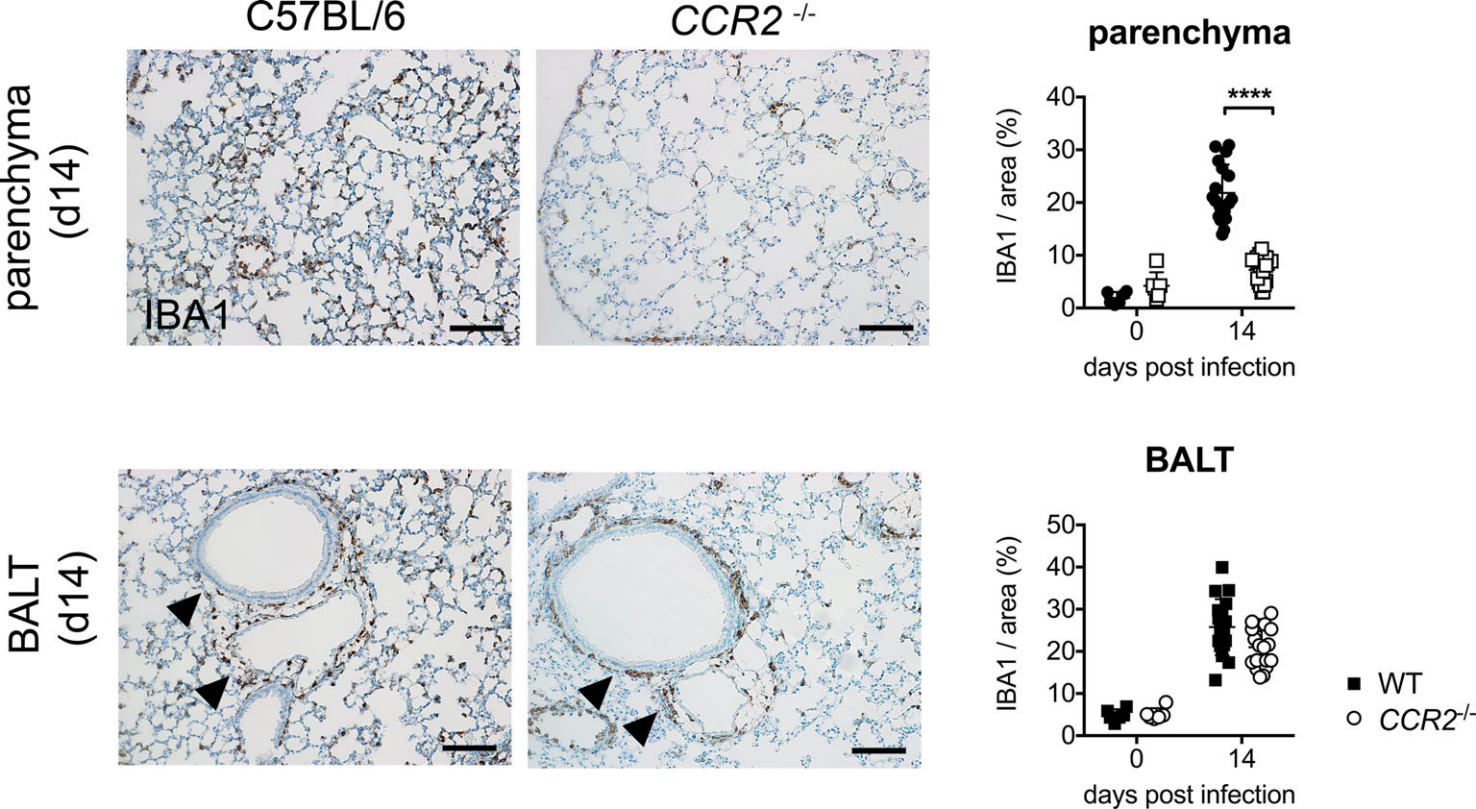
CCR2-dependent macrophage infiltration in lung parenchyma rather than peribronchial BALT. *CCR2^-/-^* and C57BL/6 mice were infected with 5,000 sfu *O. tsutsugamushi* i.d. and sacrificed on day 14 p.i. (n=3 mice per genotype). Macrophages were stained by immunohistochemistry for IBA1 in histological sections from lungs. Representative histological images are shown from parenchyma (upper row, part of the data are shown in **Figure 1D**) and peribronchial BALT areas (lower row). Scale bar: 100 µm. The IBA1^+^ area was quantified separately in parenchyma and BALT areas (6 areas per lung section from n=3 infected and n=1 uninfected mice per genotype; mean ± SD). ****p < 0.0001 by two-way ANOVA.

Last, we were interested whether CCR2 influences the histological localization of macrophages in the lung during acute *O. tsutsugamushi* infection. To that end, we performed immunohistochemistry for the macrophage marker IBA1 on FFPE sections of lung tissue. We observed that the parenchyma of wildtype mice was infiltrated by IBA^+^-positive macrophages on day 14 p.i., when the maximal bacterial concentration in lung tissue was measured. However, this parenchymal infiltration was observed to a much lesser degree in *CCR2^-/-^* mice (**Figure 6**, upper row). We quantified the IBA^+^-positive area in 6 randomly selected details from 3 mice in both genotypes by imaging software, and found that the IBA^+^-positive area in lung parenchyma was significantly larger in C57BL/6 compared to *CCR2^-/-^* mice (**Figure 6**, upper row). We also observed that this effect was restricted to the parenchyma, since the peribronchial BALT areas were similarly infiltrated by macrophages in both genotypes. Indeed, quantification showed that IBA1^+^ areas in BALT structures did not differ significantly between genotypes (**Figure 6**, lower row). However, on day 21 p.i. (data not shown), infiltration of lung parenchyma was also found in *CCR2^-/-^* mice, suggesting that CCR2-independent signals compensate this early defect and allow a later infiltration of macrophages to the pulmonary parenchyma.

Taken together, the present study investigated for the first time the mechanism of CCR2-dependent recruitment of monocytes to peripheral blood and lung tissue in a new mouse model of self-healing *O. tsutsugamushi* infection. We showed that clearance of *O. tsutsugamushi* was significantly less effective and delayed in the absence of CCR2 in the lung (and also liver), but *CCR2^-/-^* mice were eventually able to reduce the infection. CCR2 was required for mobilization of monocytes to the blood and enhanced the influx of Ly6C^hi^ monocytes, most of which developed an activated macrophage phenotype, into the lung. Unexpectedly, there was also a clear dependence of the pulmonary Ly6C^lo^ monocyte/macrophage population on CCR2, while neutrophils were unaffected in number. We could also show that early interstitial infiltration of the lung parenchyma by IBA1^+^ macrophages was CCR2-dependent, while development of peribronchitis was CCR2-independent. In contrast, expansion of Ly6C^hi^ and Ly6C^lo^ populations and bacterial clearance in the spleen were not affected by CCR2 deficiency. Our data suggest that CCR2 orchestrates the temporospatial recruitment and activation of monocytes and macrophages to the lungs, thus contributing locally to the reduction of *O. tsutsugamushi* infection.

## Discussion

Inflammatory monocytes, phenotyped as CD11b^+^ Ly6C^+^ Ly6G^-^ in mice, have been implicated in the defense against bacterial, viral, fungal and protozoal pathogens. Their emigration from the bone marrow to the circulation is mediated by interaction of CCL2 (MCP-1) with the chemokine receptor CCR2 (14), which is also highly expressed by CD11b^+^ Ly6C^+^ monocytes (47). Guided by the observations that CCL2 (MCP-1) is strongly induced by *O. tsutsugamushi* (2–4), that *Orientia* is associated with monocytes or monocyte-like cells in eschar lesions and peripheral blood (1, 6) and grows within monocytes (10), we investigated the role of CCR2 in monocyte responses in a murine model of self-healing, i.d. *Orientia* infection.

First, we detected *Orientia* in IBA1^+^ macrophages in the lung, which are an important component of pulmonary inflammation (7). While it is known that *Orientia* infects monocytic/macrophage-like cells and dendritic cells in the skin (1), the nature of its host cell involved in systemic dissemination is not known. Several studies suggested that CD11b^+^ or Ly6C^+^ monocytes, as host cells for intracellular pathogens including *Toxoplasma gondii*, *Burkholderia pseudomallei* or *L. monocytogenes*, are responsible for the pathogens’ dissemination to other organs, e.g. the brain (48–50). While we had initially suspected that inflammatory monocytes, which might later differentiate into macrophages, could mediate the dissemination of *Orientia* to organs distant from the inoculation site, this hypothesis had to be rejected with view to the equal or even increased bacterial concentrations in *CCR2^-/-^* mice in lung, liver and spleen. Given the absence of increased blood monocytosis and the onset of macrophage influx in lung tissue around day 10 p.i., it is unlikely that CCR2-dependent mechanisms play a role in bacterial clearance before that time point. However, we cannot exclude that CCR2 deficiency may influence bacterial replication by some innate mechanism in this early phase.

Several studies have underlined the importance of CCR2-dependent recruitment of inflammatory cells for the control of intracellular pathogens: *CCR2* ^-/-^ mice were significantly more susceptible to i.v. infection with *Listeria* (13), i.v. infection with *M. tuberculosis* (15), s.c. infection with WNV (16), intranasal infection with HSV (51) and *Histoplasma capsulatum* (52), and oral or i.p. infection with *Toxoplasma* (53, 54). Contrarily to these intracellular pathogens, our data show that *Orientia* is unique in that CCR2 was not required to confer protection in our intradermal infection model. As a limitation to this study, we cannot exclude that other inoculation routes (e.g. i.v.), higher infection doses or infections in male mice – male mice express higher levels of CCR2 than females in non-classical splenic monocytes (55) – would produce a different phenotype. Also, the role of CCR2 was not investigated in functional assays.

Inflammatory monocytes may influence susceptibility to infection in at least two ways: (1) by participating in antimicrobial defense, thus reducing bacterial replication, or (2) by increasing immunopathology. In our i.d. model of *Orientia* infection, we showed that CCR2 was required to reduce infection in the lung. *CCR2* ^-/-^ mice showed significantly reduced recruitment of CD11b^+^ Ly6C^hi^ and Ly6C^lo^ monocytes to the lungs, a delayed infiltration of IBA1^+^ cells to the lung parenchyma, a delayed development of the inflammatory cytokine milieu, and delayed clinical recovery. However, none of the *CCR2* ^-/-^ mice succumbed to the infection by day 21 p.i. Of note, there was no defect of bacterial clearance in the spleen, where an unimpaired expansion of CD11b^+^ Ly6C^hi^ and Ly6C^lo^ monocytes was found in *CCR2* ^-/-^ mice. The results suggest that small but eventually sufficient numbers of monocytes can be recruited in the absence of CCR2 in *Orientia* infection, either from other organs than the bone marrow, perhaps the spleen, or *via* other receptors. These CCR2-independent mechanisms of antimicrobial defense are sufficient to protect against *Orientia*.

We provide experimental evidence that the pulmonary interstitial IBA1^+^ macrophage inflammation was CCR2-dependent, while the peribronchially infiltrating macrophages appeared in a CCR2-independent fashion. These cells could be an alternative source of macrophages that later migrate into the lung parenchyma. In fact, not all tissue monocytes or macrophages that expand during infection are derived from blood monocytes that are recruited from the bone marrow *via* CCR2: A seminal study by Jenkins et al. demonstrated, in the context of a nematode infection, that macrophages can proliferate locally in the pleural cavity, independent from bone marrow, in response to IL-4 (56). Indeed, we measured a significant increase of local *il-4* mRNA transcription in the lung on day 14 p.i. in *CCR2* ^-/-^ mice, albeit later than in wildtype mice. It will therefore be of interest to study the mechanisms of bone marrow-independent macrophage inflammation in *Orientia* infection in the future, e.g. the role of IL-4-driven local proliferation or recruitment from the spleen.

It is interesting to note that the induction of Th2 cytokines appears to depend on the infection route: In the i.v. infection model of *O. tsutsugamushi* Karp that allows rapid dissemination to the organs without passing through the lymph node, transcription of *il-4* and *il-13* mRNA was found suppressed and thus interpreted as an impaired Th2 response (57). In lungs of i.d. infected mice, however, Soong et al. detected IL-13 besides TH1-related cytokines, suggesting a mixed Th1/Th2 response (58). Our data demonstrate that CCR2 deficiency did not completely shift polarization towards one pole in *Orientia* infection, but rather delayed the afore-mentioned mixed phenotype. This is different from *Cryptococcus neoformans* infection where CCR2 deficiency shifted the polarization completely from Th1 to Th2 (59). Our results show that, in the i.d. infection model, the CCR2-independent macrophage response, albeit delayed, is eventually sufficient to control *Orientia* infection in the lung.

While we initially expected that abrogation of CCR2 would reduce primarily the Ly6C^hi^ inflammatory monocytes, we found that also the Ly6C^lo^ monocytes were affected in various ways during *Orientia* infection: In CCR2-deficient animals, *Orientia* infection induced significantly lower concentrations of Ly6C^lo^ monocytes in peripheral blood and the lungs, and also the expression of MHCII and F4/80 by pulmonary Ly6C^lo^ monocytes was significantly decreased during infection in *CCR2* ^-/-^ mice.

For Ly6C^hi^ inflammatory monocytes, the role of CCR2 in regulating the circulation of inflammatory monocytes is well understood. Ly6C^hi^ inflammatory monocytes express CCR2 at high levels (44, 47, 60). The expression of CCR2 mediates their egress from the bone marrow to the blood circulation, in response to local CCL2 production by mesenchymal stem and progenitor cells which in turn respond to low concentrations of circulating TLR ligands (14, 33). We now show that during *Orientia* infection, CCR2 was required not only to elicit a systemic Ly6C^hi^ inflammatory monocyte response, but also to induce peripheral Ly6C^lo^ monocytosis and for the induction of a pulmonary Ly6C^lo^ monocyte population. Notably, induction of monocyte responses and bacterial clearance were independent of CCR2 in the spleen in our model. The spleen was shown to provide a large reservoir of undifferentiated monocytes that assemble in clusters in the cords of the subcapsular red pulp (45). From here, they can rapidly exit and deploy to inflamed tissues in a CCR2-independent fashion. These monocytes can derive from splenic common monocyte progenitors (cMoP’s), a highly proliferative cell type that may give rise to several monocyte subsets and macrophages (46). For clearance of *Orientia* from the spleen, these local splenic mechanisms appeared sufficient, without requirement to recruit monocytes from the bone marrow *via* CCR2.

There are at least two factors that could contribute to this finding: circulation and activation of Ly6C^lo^ monocytes could either be directly regulated by CCR2, or Ly6C^lo^ monocytes are derived from CCR2-dependent Ly6C^hi^ monocytes as their precursors. In support of the first mechanism, some studies show that there is low-grade expression of CCR2 on Ly6C^lo^ monocytes (44), which we also confirmed in our study. This low-grade expression of CCR2 could partially contribute to their exit from the bone marrow. More importantly however, other studies support that Ly6C^hi^ monocytes are obligatory precursors of Ly6C^lo^ cells in blood and tissue (22, 26, 43, 61). It was shown that the transition of CCR2^hi^ CX3CR1^lo^ to CX3CR1^hi^ CCR2^lo^ monocytes in response to sterile injury is mediated by local production of the cytokines IL-4 and IL-10 (22). More specifically, Yona et al. demonstrated in a mixed bone marrow chimera that the generation of peripheral Ly6C^lo^ monocytes in steady state critically depends on CCR2-competent Ly6C^hi^ cells as their immediate precursors which require mobilization from the bone marrow (43). The observation that *CCR2* ^-/-^ mice harbor circulating Ly6C^lo^ monocytes (62) could, in fate-mapping mice, be explained by an extended lifespan of Ly6C^lo^ monocytes in the absence of Ly6C^hi^ monocytes (43). It is therefore likely that in *Orientia* infection, a large number Ly6C^lo^ monocytes differentiate from their CCR2-dependent Ly6C^hi^ precursor, resulting in an impaired response of both subsets in *CCR2* ^-/-^ mice. The delayed kinetics in induction of Ly6C^lo^ compared to Ly6C^hi^ responses in *Orientia* infection support that notion.

In sum, we provide here the first mechanistic study on blood and pulmonary monocyte/macrophage responses to *Orientia* in an i.d. mouse infection model. We established that both the extent and the activation of Ly6C^hi^ and Ly6C^lo^ monocyte responses depend on CCR2 in *Orientia* infection. The bacterial clearance from the lung, the development of interstitial pulmonary tissue lesions and the development of the inflammatory milieu were significantly delayed in the absence of CCR2. Correlating with these findings, CCR2 deficiency also delayed the development of clinical symptoms, suggesting an immunopathological role for inflammatory monocytes. We thus provide here a study that demonstrates a role for CCR2 in shaping systemic and pulmonary inflammation during *Orientia* infection.

## Data Availability Statement

The datasets generated for this study are available on request to the corresponding author.

## Ethics Statement

The animal study was reviewed and approved by the Hamburg Authority for Health and Consumer Protection (no. 106/15).

## Author Contributions

MP: experimentation, data analysis, manuscript draft. CK: conceptualization and experimentation, data analysis, manuscript draft. MG: experimentation ZO: data analysis HL: conceptualization, material. BF: conceptualization, funding. JS: experimentation, data analysis. All authors contributed to the article and approved the submitted version.

## Funding

CK received funding from the von Behring-Roentgen Foundation (grant 64-0011).

## Conflict of Interest

The authors declare that the research was conducted in the absence of any commercial or financial relationships that could be construed as a potential conflict of interest.
