# Supplementary material for: CCR2 Deficiency Impairs Ly6Clo and Ly6Chi Monocyte Responses in Orientia tsutsugamushi Infection
Source: Front Immunol. 2021 Jul 5;12:670219. doi: 10.3389/fimmu.2021.670219 (PMC8287586; doi:10.3389/fimmu.2021.670219)
Supplement: Supplementary file 1 [file DataSheet_1.pdf]

Supplementary figures

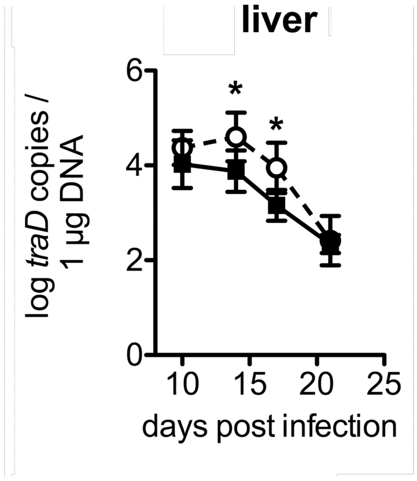

Fig. S1 Delayed clearance of *O. tsutsugamushi* in liver tissue of *CCR2*$^{-/-}$ mice

CCR2$^{-/-}$ or C57BL/6 mice were infected with 5,000 sfu *O. tsutsugamushi* in the hind footpad. Per genotype, n=3 mice were sacrificed on days 10, 14, 17 and 21, and bacterial concentrations were quantified from the liver by *traD*-specific qPCR. Data from two independent experiments were pooled; shown are means of bacterial concentrations from organs of n=2x3=6 mice per genotype and time point ± SD. * $p<0.05$ by two-way ANOVA.

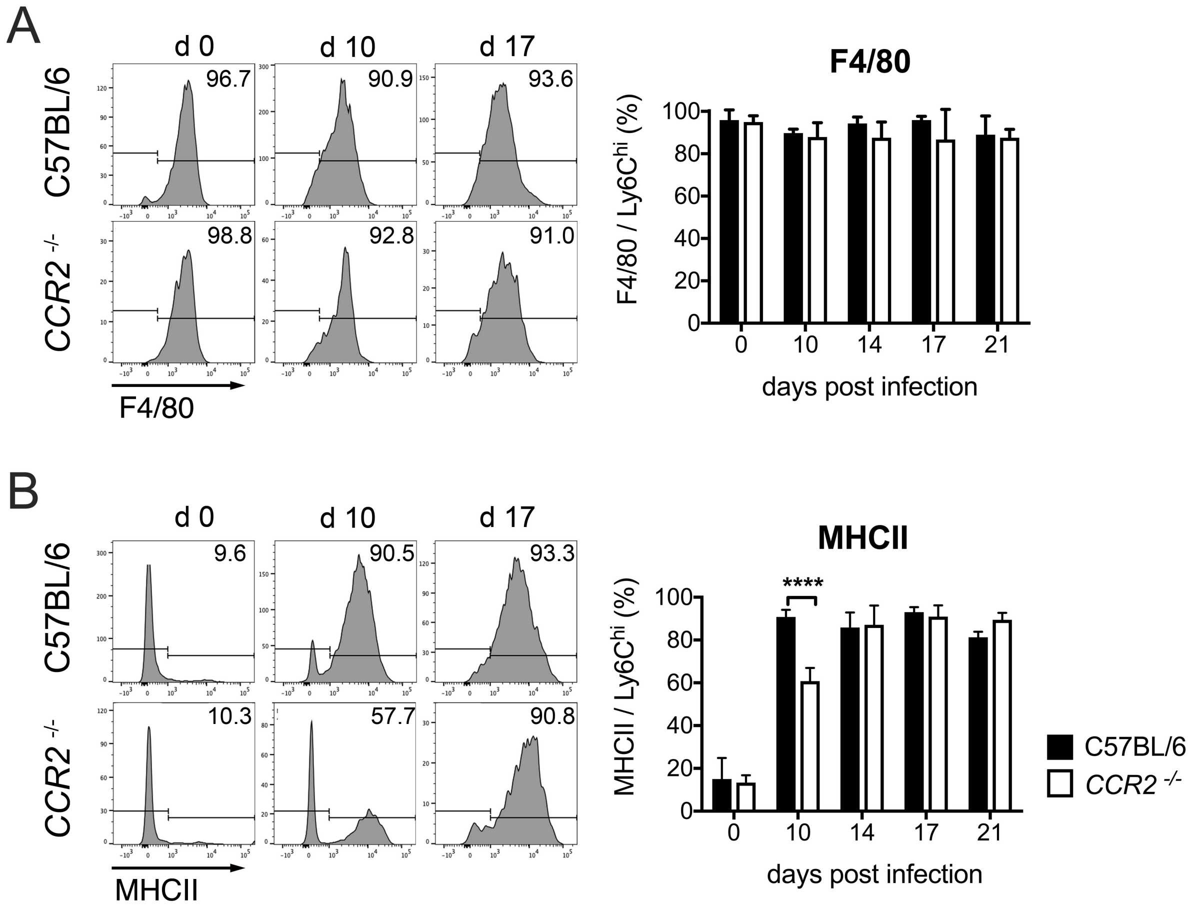

Fig. S2 Expression of MHCII but not F4/80 on *CCR2*$^{-/-}$ Ly6C^{hi} pulmonary monocytes is delayed during infection with *O. tsutsugamushi*

On CD11b^{+} Ly6C^{hi} pulmonary monocytes from infected C57BL/6 and *CCR2*$^{-/-}$ mice, the expression of F4/80 (A) and MHCII (B) was measured by FACS. Shown are percentages of positive Ly6C^{hi} cells expressing the respective marker (histograms and statistics). Data were pooled from 2 independent experiments (n=2x3=6 mice per timepoint and genotype, mean ± SD). * p<0.05; ** p<0.01; *** p<0.001 by two way-ANOVA.

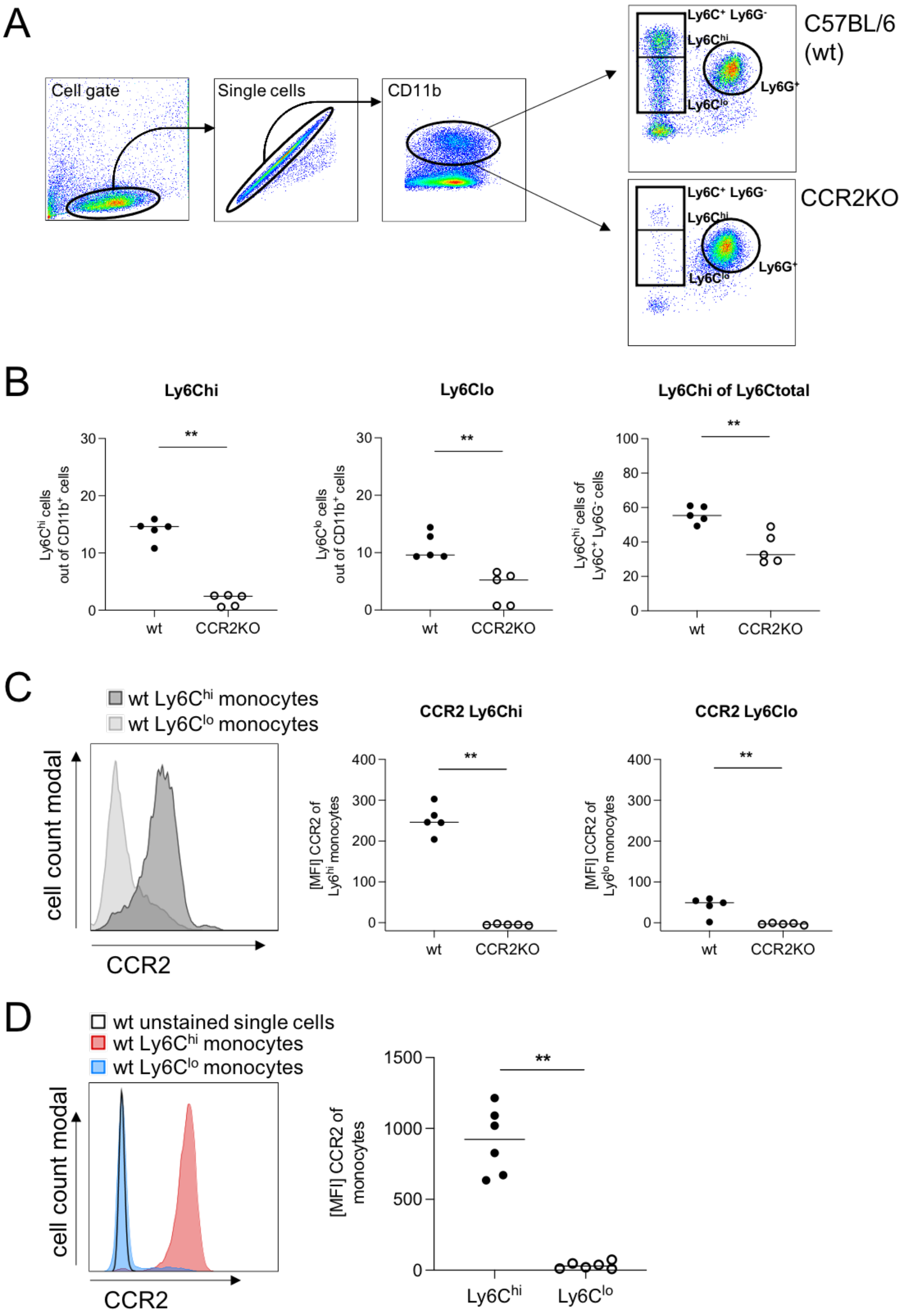

Fig. S3 Expression of CCR2 on CD11b^{+} Ly6C^{hi} and Ly6C^{lo} monocytes

PBMC from peripheral blood of C57BL/6 and *CCR2$^{-/-}$* mice were stained for CD11b, Ly6C, Ly6 and CCR2 (n=5 animals). (A) Gating strategy. (B) Among all CD11b^{+} cells, C57BL/6 mice show higher percentages of Ly6C^{hi} and Ly6C^{lo} monocytes than *CCR2$^{-/-}$* mice (left and middle panel), and higher percentages of Ly6C^{hi} per all Ly6C^{+} monocytes (right panel). (C) CCR2 expression on Ly6C^{hi} and Ly6C^{lo} monocytes (histogram for C57BL/6 mice, left panel). C57BL/6 express higher levels of CCR2 than *CCR2$^{-/-}$* mice not only in Ly6C^{hi} (middle panel) but also Ly6C^{lo} monocytes (right panel). (D) Histogram of CCR2 staining with unstained control. **, $p<0.01$ by Mann-Whitney test.

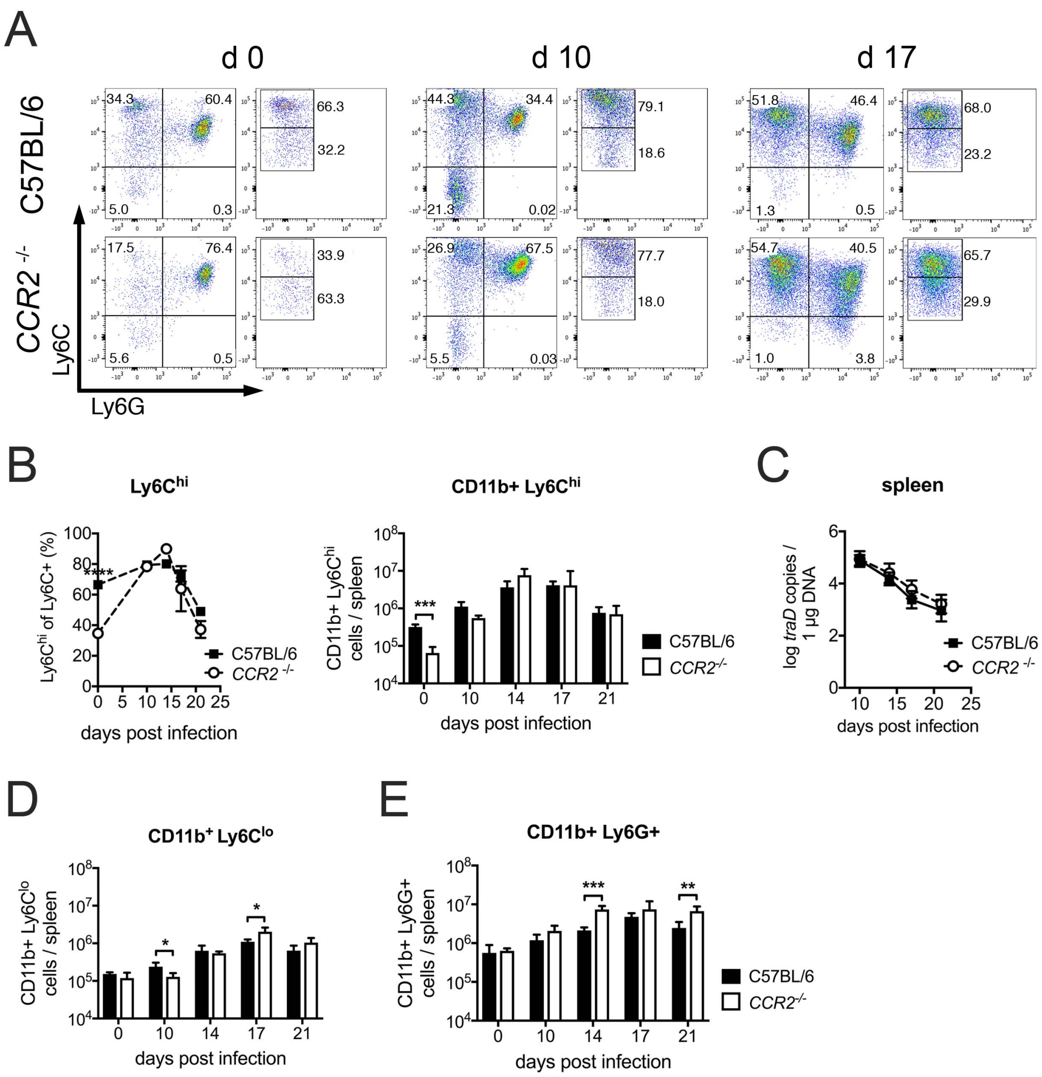

Fig. S4 CCR2 deficiency does not cause profound deficits in splenic monocyte/neutrophil responses and antibacterial defense during infection with *O. tsutsugamushi*

C57BL/6 and *CCR2*$^{-/-}$ mice were infected with 5,000 sfu *O. tsutsugamushi* in the hind footpad, and spleens were removed at days 10, 14, 17 and 21 p.i. and processed for further analysis. (A) Expression of Ly6C and Ly6G was measured by FACS on pre-gated CD11b^{+} cells (see Fig. 3). (B) Shown are percentages of positive Ly6C^{hi} of all LyC+ cells (left panel) and the entire number of CD11b^{+} Ly6C^{hi} cells per spleen (right panel). (C) On days 10, 14, 17 and 21 p.i., the bacterial concentration was measured by *traD* qPCR from spleen samples. (D) The size of CD11b^{+} Ly6C^{lo} monocyte and (E) CD11b^{+} Ly6G^{+} granulocyte populations was analyzed from FACS data. Data in C were from two independent experiments (n=6 mice per genotype and timepoint, mean ± SD); data in A,B,D,E were from one experiment (n=3 mice per genotype and timepoint, mean ± SD). * $p<0.05$; ** $p<0.01$; *** $p<0.001$, **** $p<0.0001$ by two way-ANOVA.
